# Strategies to reduce hyperglycemia-related anxiety in elite athletes with type 1 diabetes: A qualitative analysis

**DOI:** 10.1371/journal.pone.0313051

**Published:** 2025-01-17

**Authors:** Alexandra Katz, Aidan Shulkin, Marc-André Fortier, Jane E Yardley, Jessica Kichler, Asmaa Housni, Meryem K. Talbo, Rémi Rabasa-Lhoret, Anne-Sophie Brazeau

**Affiliations:** 1 Faculté de Médecine, Université de Montréal, Montréal, Québec, Canada; 2 School of Human Nutrition, McGill University, Montreal, Québec, Canada; 3 Institut de Recherches Cliniques de Montréal, Montréal, Québec, Canada; 4 École de kinésiologie et des sciences de l’activité physique, Université de Montréal, Montréal, Québec, Canada; 5 Department of Psychology, University of Windsor, Windsor, Ontario, Canada; 6 Service d’endocrinologie du Centre Hospitalier de l’université de Montréal (CHUM), Montréal, Québec, Canada; King Faisal University, SAUDI ARABIA

## Abstract

**Objective:**

Managing blood glucose levels is challenging for elite athletes with type 1 diabetes (T1D) as competition can cause unpredictable fluctuations. While fear of hypoglycemia during physical activity is well documented, research on hyperglycemia-related anxiety (HRA) is limited. HRA refers to the heightened fear that hyperglycemia-related symptoms will impair functioning. This study investigates current strategies employed to mitigate HRA during competition and the development of alternative approaches.

**Research design and methods:**

Elite athletes with TID, aged >14 who self-reported HRA during competition were recruited. Elite athletes were defined as individuals exercising >10 hours per week whose athletic performance has achieved the highest competition level. 60 to 90-minute virtual semi-structured interviews were analyzed using an Interpretative Phenomenological Analysis.

**Results:**

Ten elite athletes with T1D (average age 25 ± 3 years; T1D duration 12 ± 8 years; number of competitions per year 27 ± 19; training time per week 12 ± 6 hours) reported the strategies they currently use to mitigate HRA. These strategies include managing insulin and nutrition intake, embracing social support networks, using technology, practicing relaxation techniques, establishing routines, performing pre-competition aerobic exercise, and maintaining adequate sleep hygiene. Several additional approaches that could be implemented were identified including establishing targeted support networks, developing peer-reviewed resources on HRA, ensuring support teams have sufficient tools, and improving existing technology.

**Conclusions:**

Elite athletes with T1D use physiological and psychological strategies to mitigate HRA during competition. This finding highlights the need for increased support and education for these athletes, and advancements in technology. A multidisciplinary approach involving healthcare professionals, athletic staff, and peer mentors could help integrate personalized anxiety management and diabetes care strategies into training regimens, enhancing both mental resilience and performance outcomes for athletes with T1D.

## Introduction

Individuals with type 1 diabetes (T1D) compete at the highest level of their sports, including winning Olympic gold medals and becoming professional athletes [1]. However, participating in physical activity (PA) poses considerable challenges for athletes with T1D, primarily due to its multifaceted influence on glycemia [2–5]. T1D is an autoimmune disorder in which the insulin-producing beta cells of the pancreas are destroyed, leading to a significant reduction or complete cessation of insulin production. Incorrect management of blood glucose (BG) modifiers, including insulin, pre-competition meal composition, exercise duration and intensity, stress levels, and sleep quality can lead to dangerous fluctuations in BG levels which can result in hyper- or hypoglycemia [6, 7].

Most people with T1D prioritize avoiding hypoglycemia during PA as it can compromise performance [8] due to lack of concentration, headaches, dizziness, and confusion [9]. Conversely, athletes may also worry about hyperglycemia, which can also reduce performance through debilitating symptoms such as shortness of breath, thirst, slower reaction time, and blurred vision [3, 10].

Fear of hypoglycemia during PA is well documented [11], however, research on hyperglycemia-related anxiety (HRA) is limited. HRA refers to the heightened fear or worry that hyperglycemia-related symptoms will impair functioning [10]. Unlike Generalized Anxiety Disorder (GAD), which involves persistent and excessive worry across multiple aspects of life, HRA is specifically tied to concerns over hyperglycemia-related symptoms.

Hyperglycemia is often considered a chronic condition, with its acute symptoms typically causing minimal disruption to daily life [12]. However, any element that hinders competition performance in elite athletes is a significant concern [10]. Various factors, including competition-associated stress may cause hyperglycemia [2, 13, 14]. A position statement from the *National Athletic Trainers’ Association* [14] acknowledges that high anticipatory stress can elevate counterregulatory hormones, such as adrenaline and cortisol, potentially increasing BG before and during competition [11]. This specifically affects elite athletes, as they face increased amounts of performance-related stress [15]. This can create a vicious cycle where competition-related stress exacerbates HRA, and in turn, HRA amplifies the stress and anxiety associated with performance.

Current recommendations for diabetes management are not adapted to the specific physiological and psychological demands of competition [13, 16]. For example, athletes who expect a BG rise before competition are more prone to taking preventive measures (e.g., injecting more insulin, and limiting pre-PA carbohydrate intake) [2]. This highlights a gap in strategies specifically designed to address these nuanced needs. A recent case study suggests a need for tailored treatment programs for elite athletes with T1D who experience HRA [10], but to our knowledge, no other studies exploring this topic are available in scientific literature. Therefore, this study aims to understand the impact that HRA has on competition and diabetes management among elite athletes with T1D. Additionally, it explores the strategies used by these athletes to prevent HRA during competition and maps out additional approaches that they can use to optimize BG management.

## Methods

### Study design, participants & recruitment

This cross-sectional qualitative analysis was conducted following the Consolidated Criteria for Reporting Qualitative Research checklist [17]. Ethics approval was obtained by the Centre Hospitalier de l’Université de Montréal ethics committee (2024–11786 (23.156)). Potential participants were screened for eligibility, consented to participation, filled out a demographic questionnaire, and registered for an interview via Research Electronic Data Capture (REDCap) [18].

Individuals were eligible to participate in this study if they were elite athletes ≥14 years old, had a T1D diagnosis for ≥1-year, self-reported HRA prior and/or during competition, used intensive insulin therapy, and spoke English or French. Participants were excluded if they had a diagnosis of GAD or a score of >10 on the GAD 7-item scale [19]. Elite athletes were defined as individuals exercising >10 h/week whose athletic performance had achieved the highest competition level (definition based on [20]). For the purpose of this study, HRA was defined as: (1) excessive worry, fear or distress solely in relation to hyperglycemia prior and/or during competition; (2) occurring frequently prior and/or during competition for at least 6 months; (3) difficult to control; (4) associated with symptoms of hyperglycemia; (5) causing significant distress or impairment prior and/or during competition; (6) the disturbance is not attributable to the physiological effects of a substance or a diagnosed medical condition/psychological disorder. This definition was inspired by the GAD criteria from the DSM-5 [21].

Recruitment was conducted via word-of-mouth, at the Montreal Clinical Research Institute (IRCM) clinic, and by internet postings (e.g., newsletters, websites, social media, etc.) from November 8^th^ to December 11^th^, 2023.

### Interviews

Semi-structured individual interviews were scheduled for 60–90 minutes on Microsoft Teams and were conducted in English or French. Information obtained through REDCap was verified for each participant. To enhance the understanding of participant perspectives and acknowledge the potential impact of the researchers’ positionality, the research team employed bracketing, defined as the systematic suspension of preconceptions and biases during data collection and analysis [22]. The researchers documented their initial assumptions and beliefs about the study topics before conducting interviews and revisited these notes throughout the analysis process to ensure they were not influencing data interpretation. Additionally, the researchers maintained ongoing reflexive notes throughout the interviews and analysis to track their evolving understanding and to safeguard the integrity of the findings. By employing these techniques, the researchers maintained transparency and reflexivity, ensuring that the analysis remained grounded in the participants’ perspectives while also accounting for the influence of the researchers’ positionality.

**Fig 1:**
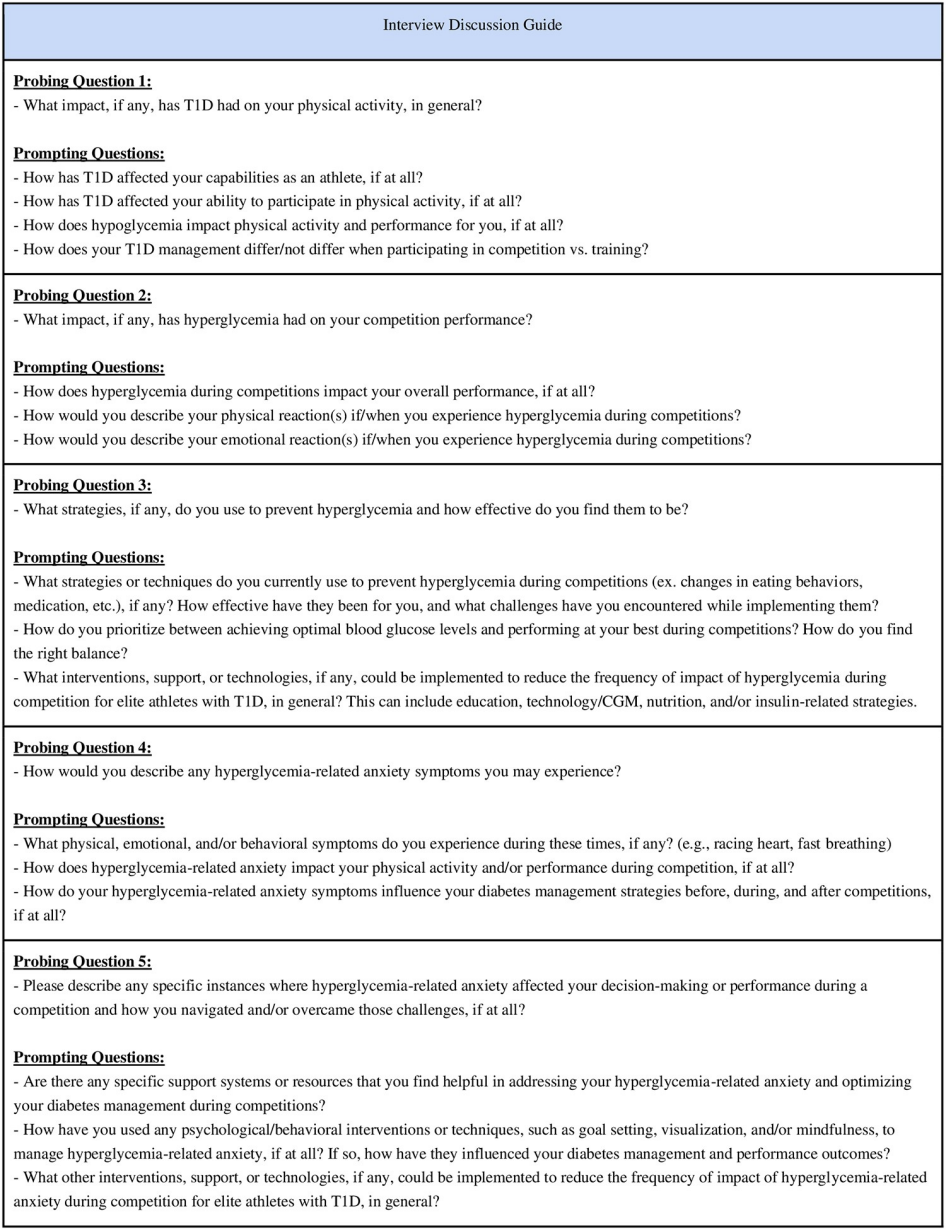
Interview discussion guide.

A research team member followed an interview discussion guide (Fig 1) to direct the open-ended conversation using five probing and additional prompting questions derived from the *Hyperglycemia Avoidance Scale* [23]. The guide was reviewed by a multidisciplinary team including dietitians, an endocrinologist, a clinical psychologist, a kinesiologist, and a patient partner. Before the interview, participants were given the probing questions to reflect on. Additional resources were available for mental health support if necessary.

### Transcript and analysis

Interviews were recorded and transcribed using Microsoft Teams. A research team member (MA.F.) verified and anonymized the transcriptions. Recordings were deleted to preserve confidentiality. French meeting transcriptions were translated to English using a forward-backwards translation method [24].

Transcriptions were uploaded to MAXQDA (version 24, VERBI GmbH, Berlin, Germany) [25]. The beta AI Assist function was used to generate preliminary codes. Using an Interpretive Phenomenological Analysis approach, two independent researchers (A.K. and A.S.) reviewed and refined the codes to create the codebook, which was applied to the entire dataset. Overarching concepts, relationships, or sequences among the themes were identified and analyzed.

A detailed essence description of participants’ experiences and main themes was created. Study participants verified the essence description to complete the member-checking process. Once validated, revisions were made based on feedback. All comments provided by the participants during this process were incorporated into the analysis, ensuring that their personal experiences were accurately reflected. Participants were provided with a $50 CAD electronic Amazon gift card or a donation in their name to a diabetes-specific charity of their choice.

## Results

Ten elite athletes (5 males, and 5 females) were interviewed from November to December 2023. Participants had an average age of 25 ± 3 years, had a T1D duration of 12 ± 8 years, participated in 27 ± 19 competitions per year, and performed 12 ± 6 hours of training per week. Four participants were tier 3, and six participants were tier 4 athletes (based on the Classification Framework developed by McKay et al (2022) [26]). Four participants used continuous subcutaneous insulin infusions (CSII), and six participants used multiple daily injections (MDI). All athletes used real-time continuous glucose monitoring (rtCGM) and its alerts/alarm feature in the last year. They originated from Canada, the United States, or the United Kingdom and self-reported as Caucasian. The athletes engaged in various sports, including speed skating, cross-country/track running, ice hockey, rugby, volleyball, basketball, and baseball. Participant characteristics are summarized in Table 1.

**Table 1:** Participant characteristics.

Participant	Age	Sex	Tier^a^	Diabetes Duration (years)	Past Ketoacidosis Episode	Insulin Administration Method	Competitions per Year (number)	Average Training Time per Week (hours)^b^
1	29	Male	4	2	No	MDI	10	25
2	24	Female	3	6	No	MDI	7	8
3	27	Female	4	7	Yes	MDI	30	6
4	25	Female	3	21	No	MDI	15	9
5	30	Female	4	22	Yes	CSII	40	8
6	29	Male	3	19	Yes	CSII	24	13
7	22	Male	4	3	Yes	MDI	36	12
8	23	Male	4	18	No	CSII	25	18
9	22	Female	3	21	No	CSII	10	7
10	22	Male	4	5	Yes	MDI	75	18

CSII, continuous subcutaneous infusion; MDI, multiple daily insulin injections

^a^Tier classification was based on the Classification Framework developed by McKay et al (2022) [26]. Tier 5: World Class athletes that have Olympic or world medals, are record holders and/or ranked top 3 in the world for their respective sport. Tier 4: Elite/International level. Athletes are competing at the international level individually or within their national team and/or ranked between 4–300 within their respective sport. Tier 3: Highly Trained/National Level. These athletes are competing at the national level and/or state leagues/tournaments. Tier 2: Trained/Developmental. These athletes perform at the local level, are training ∼3 times per week, and train with the purpose of competing. Tier 1: Recreationally Active individuals. Participants within this tier meet World Health Organization minimum activity guidelines of 150–300 minutes of moderate-intensity activity or 75–150 minutes of vigorous-intensity activity a week. Tier 0: Sedentary individuals. Participants who do not meet minimum activity guidelines.

^b^Training time per week excludes any additional exercise performed outside of sport-specific training or competition.

## Hyperglycemia in elite athletes with T1D

### Hyperglycemia symptoms

Participants reported experiencing a variety of hyperglycemia symptoms during both training and competition. These included irritability, muscle cramps, impatience, agitation, attention deficiency, excessive thirst, blurred vision, delayed reaction time, frequent urination, fatigue, abdominal pain, and shortness of breath. Elite athletes highlighted that certain symptoms had a more significant impact due to the rigorous demands of their training and competitive environments. For instance, delayed reaction time proved particularly detrimental during critical moments in competitions requiring quick reflexes. Maintaining focus over extended periods was notably challenging, and exertion levels exacerbated feelings of fatigue, affecting performance. Additionally, severe muscle cramps were a common issue that often required immediate attention to prevent complications during competition.

### Stress-induced hyperglycemia

Athletes reported a relationship between stress and hyperglycemia, particularly before and during competitions. A participant stated, “Before [competition], I have so much adrenaline that my [BG levels] skyrocket,” and during competition, “No matter what I do, my [BG levels] go up to 20 or 25.” These descriptions suggest that anticipatory stress and competitive environments contribute to acute BG elevations. Additionally, a participant noted, “I’m stressed because I see my BG not being optimal […], and then I’ll be even more stressed […] So, I’m more reactive than proactive.” This demonstrates a vicious cycle where competition-related stress intensifies HRA, and in turn, HRA further heightens the stress and anxiety linked to performance.

### Hyperglycemia-related anxiety

Beyond the physical symptoms, hyperglycemia led to constant stress that affected the athletes’ mental well-being, leading to HRA, “On a more psychological level, it’s a constant stress if [my BG] is not perfect or at the number I would want it to be for optimal performance.” The chronic nature of this stress was described as “in the background of your mind all the time” prior and during competition. Additionally, fear of underperforming due to hyperglycemia added another layer of psychological strain, “In hyperglycemia, I don’t feel good. I don’t want to feel unwell during my sport and perform poorly or make mistakes that I wouldn’t normally make.” Feeling uncomfortable discussing it with teammates who cannot relate worsened the psychological impact for many, “You can’t really talk to a teammate about [HRA]. It’s not like a pulled groin or a torn ligament.” Athletes expressed how stress affects their BG variation, emphasizing the need for targeted strategies. These strategies should aim to manage stress, thereby improving glycemic management and overall performance.

### Strategies currently used to prevent HRA

Elite athletes discussed their strategies for reducing hyperglycemia during competition, therefore limiting HRA.

#### Insulin and nutrition

Athletes mentioned increasing basal insulin before a competition to prevent hyperglycemia. A participant stated, “I went from taking five units to eight units leading into that [competition]” to ensure normalized BG levels. Athletes also discussed increasing bolus insulin mid-game, as pre-competition adjustments were insufficient, highlighting the challenges of predicting insulin needs during competition, “I take more insulin because sometimes [my BG] continues to rise [mid-competition].”

Athletes stressed the importance of pre-planning meals and maintaining dietary consistency while being mindful of the glycemic index of foods. Many opted for complex carbohydrates to prevent rapid elevations; others focus on the timing and order of nutrient intake to control digestion rates, “You’re not getting that [BG] spike all at one time.” Athletes also emphasized the importance of staying well-hydrated, highlighting its impact on mitigating their BG variability.

#### Social support network

Engaging with healthcare professionals (HCPs) was highlighted as crucial for minimizing HRA, “My family doctor makes appointments with me every two weeks and we go through all my [rtCGM] data.” The involvement of physicians was also discussed, with a participant sharing that their endocrinologist provided them with potential strategies based on another of their patients’ experiences, “My endocrinologist is [a professional athlete’s] endocrinologist […] I know that on the bench, he has a tablet that is directly connected to his [rtCGM]. As soon as he comes off the ice, he sees his BG automatically.” Overall, athletes described the importance of consultations with trusted HCPs to develop personalized strategies to optimize BG levels, thus reducing HRA. These consultations not only provide medical expertise but also foster confidence and a sense of security.

Furthermore, the study highlighted the support of dietitians and mental performance coaches. Dietitians provided tailored advice on nutrient timing, optimized pre-competition fueling strategies, and post-exercise recovery nutrition to support both glycemic control and athletic performance. Mental performance coaches contributed to psychologically managing HRA, particularly in navigating competitive stress challenges. Mental performance coaches help athletes build self-confidence, enhance focus, and prepare for diabetes-related challenges during competition through techniques like visualization and scenario-based rehearsals. None of the athletes mentioned that their training staff provided assistance or had an adequate understanding of T1D.

Family and friends were also recognized as crucial support systems, providing emotional support and practical assistance in diabetes management, “My parents are a good outlet. […] They get all my CGM data on their phone. If I see [my mom] text me at […] 11:00 pm I’m thinking, ‘I need to check my BG.’” These support systems contributed significantly to the athletes’ well-being, thereby reducing HRA.

Additionally, peer support emerged as a valuable strategy, “One of my friends has T1D and he’s a lifesaver. He gives me tips.” The exchange of insights among T1D peers provided unique support, as athletes drew on shared experiences to gain practical advice and emotional encouragement.

The study also acknowledged that following and interacting with influencers, celebrities, and professional athletes, who share their T1D experiences on social media helped to reduce HRA, “[A professional athlete] also has T1D, and he posts on Instagram, […] sometimes I think, ‘Oh, I could try that.’”

#### Technology

Athletes unanimously highlighted rtCGMs’ importance in improving their BG level awareness and adjustments during competitions. They stated, “It can help during competitions to predict how my [BG levels] will go” and “Constantly having [rtCGM] at my fingertips, it can tell me what I’m at and if I need to make any adjustments.” Two athletes mentioned that integrating rtCGM alerts into their daily routines was a crucial tool for preventive adjustments, allowing them to address BG variations before they become problematic.

Athletes highlighted the benefits of CSII for adjusting basal rates and making real-time adjustments, especially during training and competition, “I use a [CSII], and it really helps me […] If you’re able to alleviate some decisions, that to me is very impactful to my psychological and physical well-being.” Furthermore, the integration of rtCGM with CSII technology emerged as a notably effective diabetes management approach. This combination addresses the dynamic interaction between CSII and rtCGM, offering athletes enhanced control and flexibility in managing their diabetes during various activities.

#### Psychological preparation

Athletes highlighted the role of meditation in managing HRA, “I try to focus a lot on the mental side of meditating pre-game to avoid that huge adrenaline spike.” They specifically mentioned meditation as crucial for clearing the mind the night before their competition, “I always meditate the night before [competition]. It helps [me] relax because there’s a lot of anxiety about [competing].”

Additionally, athletes described the positive effects of mental preparation on BG stability, “I did mental preparation this year for the first time, and […] my BG levels were much more stable.” This effect suggested a potential link between mental preparedness, reduced stress, and improved BG levels. They also stated, “Being more prepared, being more in control, being more comfortable, I’m less stressed, so my BG rises less.”

Athletes also highlighted the efficacy of goal setting in mitigating HRA. However, they cautioned against having too many goals, as it can intensify anxiety and stress, “When you have 14 [goals], it can seem overwhelming and amplify anxiety. When it’s just one, two or three [goals], it’s a little bit easier.” Additionally, the importance of setting realistic goals was mentioned, “If you set unrealistic goals […], it’s really easy to get let down.”

Finally, athletes noted the importance of focusing on performance and avoiding additional stress, “I concentrate on my performance and forget about diabetes during that time.” This performance-centric mindfulness approach helps them manage BG levels more effectively. Having self-awareness about the current circumstances is also essential to mitigate anxiety, “I’m high, I’m dealing with it, and trusting […] I will come down […] I can’t do anything other than focus on my game.”

#### Routines

On competition days, athletes emphasized the importance of pre-established routines in better predicting BG variability, which directly helped mitigate HRA. By adhering to a consistent routine, athletes gained a sense of control over their BG levels, reducing the uncertainty that typically triggers anxiety. As one athlete explained, “Having a game plan, an established routine before a game, helps me.” This athlete also details their game-day routine, “On game days, I have a pre-established routine. I […] always have the same breakfast and make the same smoothie with the same quantities.” The athlete’s focus was to minimize variability, which in turn reduced the emotional stress of unpredictable BG fluctuations. By fostering predictability, athletes felt more confident in their ability to manage their diabetes, leading to a reduction in HRA and improving their overall performance.

#### Aerobic exercise

Athletes highlighted the significance of engaging in aerobic exercise before competition as a valuable HRA management tool. One athlete mentioned incorporating long walks after meals and before a competition, perceiving a potential decrease in stress and subsequently BG levels, “If you’re high, [it helps to] do a prolonged aerobic warm up. That’ll probably bring you into range.” The effects of aerobic exercise on the body were noted as significant, as it not only helped regulate BG levels but also reduced the anxiety associated with unpredictable fluctuations.

Another athlete timed their workouts with periods in which they typically had lower BG levels, such as mornings. By aligning aerobic exercise routines with biological patterns, the athlete leverages their body’s natural responses to optimize BG control. One athlete stated that by knowing their “[BG] patterns, I try to work around them and use them to my advantage.” By tailoring their exercise routines to their personal BG patterns, athletes effectively minimized HRA while ensuring they were well-prepared for competition.

#### Sleep

Athletes noted that insufficient or unrestful sleep could impair performance, consequently increasing anxiety and contributing to BG elevations the following day, “If I don’t get enough sleep or I’m not properly rested, I find that [my BG] can spike.” Implementing structured bedtime routines to enhance sleep quality was also reported. They added, “I’ll have herbal tea before I jump in the shower […] then I’ll stretch and meditate […] that’s been a big help for me.” Late-night schedules and deviations from regular sleep patterns were identified as factors contributing to BG management challenges.

Athletes reported using natural sleep aids such as melatonin and magnesium to increase relaxation and improve sleep quality. However, challenges were identified such as experiencing morning fogginess, which affected their performance during early-morning competitions, “Our [training] starts at 9:00 am, so I’m up at 5:30 to 6:00 am. The melatonin [..] has me in a fog through the morning. You want to feel sharp […], you want to feel ready, especially when [every second] matters.” This underscores the importance of considering the timing and potential side effects of sleep aids in the context of athletic schedules.

### Development of strategies to support elite athletes with HRA

#### Social support network

Given that there are few elite athletes with T1D, building social support networks can be challenging. A participant stated, “If I knew another athlete who has really tight control, like [a professional athlete], I’m sure they don’t have the same problems as me because he has a team of medical professionals.” This statement demonstrates the need for support networks to provide T1D athletes with a platform for shared experiences and insights. Another participant highlighted the importance of creating, “A community where you can exchange […] with people who live the same reality as you […] I think that’s something that might be missing.”

Additionally, providing family and friends with credible information may improve their support, “Your family members […], they may not understand [TID].” The athlete added that developing support strategies for family and friends is essential to bridging existing gaps.

Further, teams and athletic organizations’ support staff should have access to targeted education to enhance their understanding of diabetes management, create an environment conducive to the successful integration of athletes with T1D, and reduce stigma. A participant stated, “It often happens that coaches ask me if I manage [my T1D] well. They don’t necessarily ask more questions. I think it comes from ignorance […] it’s not that they don’t care; it’s that they don’t know.” This comment underscores the need for education and training programs for coaching and support staff to ensure a comprehensive understanding of diabetes. They added, “It would help to know that the coaches are more aware of it. Maybe discussing it after a [competition] to know, ‘Okay, they understand that hyperglycemia had an impact on my performance,’ or ‘It’s a challenge in my sport performance.’” Athletes preferred training staff to understand T1D instead of taking an active role in their management, “The support that, someone like a trainer could provide would be having certain tools accessible [to understand T1D] rather than […] offering management tips.”

HCPs play an essential role in guiding athletes on diabetes management strategies and providing tailored advice. However, few HCPs have knowledge about the unique needs of elite athletes with T1D, let alone about HRA in this population.

#### Technology

Many athletes desired systems that automate insulin adjustments based on factors like exercise and carbohydrate intake. These technologies have the potential to impact psychological and physical well-being significantly, “It’d be great to have a closed-loop system where I don’t have to put in how many carbs I’m eating. Or, like, if I’m working out, it’s just able to adjust my insulin values.” However, current technologies often fall short of meeting their expectations. Furthermore, the affordability of existing technologies remained a concern, “I find [rtCGMs] to be a technology that would be worthwhile, but it’s expensive.”

Additionally, the discomfort and lack of trust associated with current CSII designs were highlighted. Some athletes refrained from using CSII due to concerns about the devices being large, bulky, and potentially unreliable, “I don’t feel comfortable playing with it, with wires […] I just wouldn’t trust it to not fall off.” Thus, redesigning CSIIs to meet elite athletes’ needs may make them more appealing.

**Fig 2:**
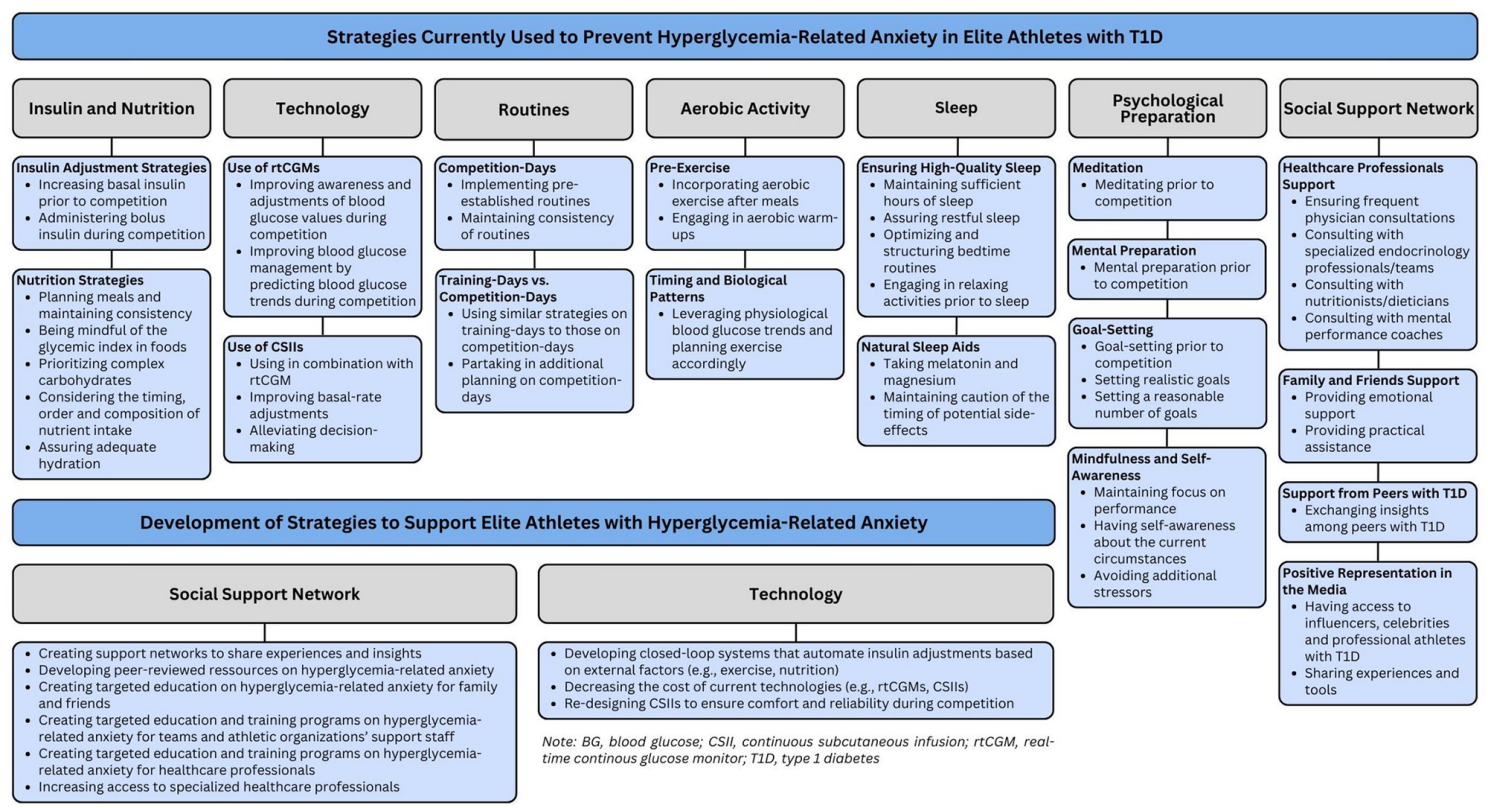
Current and future strategies to support elite athletes with T1D and HRA. Note: BG, blood glucose; CSII, continuous subcutaneous infusion; rtCGM, real-time continuous glucose monitor; T1D, type 1 diabetes.

Strategies are summarized in Fig 2.

## Discussion

This qualitative study is the first to explore HRA in elite athletes with T1D. Athletes currently use numerous strategies to reduce HRA, however, they are often insufficient. A recurring theme was the critical role of maintaining adequate BG levels to reduce HRA risk. Athletes highlighted the impact of competition on hyperglycemia due to related physical and emotional stress likely increasing stress hormones and glycemia [27–31]. While guidelines recommend checking BG frequently to understand its direction and magnitude of change [14, 16], this strategy is not always easy to implement during competition.

Consistent with previous findings [31, 32], trial-and-error approaches were prevalent among athletes with T1D. The limited documentation of tactics leaves elite athletes with few strategies [30] and a desire for additional resources. These resources could include peer mentoring, support from training staff, and the use of medical devices [30]. Moreover, athletes expressed challenges associated with CSII usage during exercise, including physical interference with sporting activities, a sentiment expressed elsewhere [6].

In Riddell et al (2020) [6], a few strategies were outlined for competitive athletes with T1D who experience competition-related hyperglycemia. These strategies include cognitive techniques, pre-competition aerobic exercises, and administering temporary basal rate increases or partial bolus insulin to correct hyperglycemia. However, they did not address HRA, similar to other scientific literature. Further, our participants’ experiences align with the suggestion from the *National Athletic Trainers’ Association* [14] regarding consulting physicians for increased basal rates or small insulin boluses during periods of exercise-induced hyperglycemia. However, there are no evidence-based guidelines that physicians can use to guide their patients in this population. Thus, there is a need to develop resources to support elite athletes with T1D [16], particularly in subject areas such as HRA. Studies have shown that having access to specialized healthcare resources that are co-developed with patients can increase confidence in diabetes management [33, 34]. Thus, future efforts should focus on developing education and awareness programs tailored to the unique needs of elite athletes with T1D, addressing the psychological and physiological challenges associated with competition-related hyperglycemia. Future strategies should be adaptive, accommodating each athlete’s unique diabetes management needs [31].

These strategies could be incorporated in real-world or clinical settings by involving sports teams, athletic trainers, and physicians in the creation of individualized plans that address both the physical and psychological challenges faced by athletes. Anxiety management techniques, such as mindfulness, relaxation exercises, and cognitive-behavioral strategies, can be tailored to the specific stressors of each sport and integrated into training regimens. For example, athletic trainers could incorporate anxiety-reduction exercises into regular training sessions, helping athletes mentally and physically prepare for competition. Endocrinologists could provide specialized guidance on adjusting insulin regimens during competition, using rtCGM data to inform real-time insulin adjustments. Additionally, fostering a supportive environment where athletes feel comfortable discussing their experiences with HRA could facilitate early intervention and continuous emotional support. Peer mentorship programs, where athletes with T1D offer practical and emotional guidance to one another, could further improve HRA management. A multidisciplinary approach involving healthcare professionals and athletic staff would not only enhance diabetes management but also contribute to better mental resilience, overall well-being, and performance outcomes for athletes with T1D. Future research should explore the practical application of these strategies and assess their effectiveness in real-world settings.

Our study has several notable strengths and limitations. The qualitative interviews inherently introduce a recall bias into the study. However, we implemented bracketing and member checking to increase reliability. The exclusion of participants with GAD limits this potential confounding variable, as individuals with pre-existing anxiety disorders may exhibit heightened responses to stressors unrelated to diabetes management, potentially skewing the results. The broad spectrum of sports represented can be seen as a strength or a weakness; while it allowed us to gain a comprehensive understanding of HRA-related consequences and strategies, it limited the detailed exploration of specific challenges within each sport. Despite the small sample size of 10 participants, thematic saturation was achieved after approximately 5 or 6 interviews, with no new themes emerging in the subsequent interviews. This suggests that the sample size, while limited, was adequate for our qualitative analysis. Our study population, predominantly including Caucasian individuals from developed nations, may limit the generalizability of the results. Additionally, we did not specifically analyze the potential impact of insulin delivery methods (CSII vs. MDI) on HRA, which could introduce temporal factors affecting performance. Future research should explore the role of insulin delivery methods to better understand their influence on anxiety patterns, self-monitoring practices, and performance outcomes in elite athletes with T1D. Furthermore, distinguishing hyperglycemia-related symptoms from fatigue-related symptoms presents a methodological challenge. While our study assumes consistency and accuracy in participants’ self-reports, the physiological demands of elite-level training and competition may lead to symptom overlap, potentially confounding interpretations of HRA. Future research should incorporate objective measures such as rtCGM data and validated questionnaires to address these limitations.

## Conclusion

In conclusion, addressing HRA in elite athletes with T1D involves refining current strategies, developing personalized tools, bridging support system gaps, and enhancing education for athletes, training staff, peers, and healthcare professionals. Both physiological and psychological strategies are crucial in managing HRA, as our findings emphasize the need for tailored approaches that address both aspects. Future studies should explore the efficacy of the strategies outlined in our findings, for example, exploring the effectiveness of peer support groups for HRA in competitive sports and conducting randomized trials to test new technologies. Developing tailored resources and support systems will better equip HCPs and athletic staff to address the multifaceted needs of elite athletes with T1D, ultimately improving both their performance and overall well-being.
